# Recent Applications of PLGA in Drug Delivery Systems

**DOI:** 10.3390/polym16182606

**Published:** 2024-09-14

**Authors:** Jie Yang, Huiying Zeng, Yusheng Luo, Ying Chen, Miao Wang, Chuanbin Wu, Ping Hu

**Affiliations:** 1 Department of Burns & Plastic Surgery, Guangzhou Red Cross Hospital, Faculty of Medical Science, Jinan University, Guangzhou 510006, China; msjieyang@163.com; 2 College of Pharmacy, Jinan University, Guangzhou 510006, China; zenghy828@163.com (H.Z.); chuanbin_wu@126.com (C.W.); 3 International School, Jinan University, Guangzhou 510006, China; 4 Guangdong Institute for Drug Control, NMPA Key Laboratory for Quality Control and Evaluation of Pharmaceutical Excipients, Guangzhou 510660, China; chenying@gdidc.org.cn (Y.C.); gdidc_wangmiao@gd.gov.cn (M.W.)

**Keywords:** PLGA, drug delivery system, polymer, microparticle, nanoparticle, health care

## Abstract

Poly(lactic-co-glycolic acid) (PLGA) is a widely used biodegradable and biocompatible copolymer in drug delivery systems (DDSs). In this article, we highlight the critical physicochemical properties of PLGA, including its molecular weight, intrinsic viscosity, monomer ratio, blockiness, and end caps, that significantly influence drug release profiles and degradation times. This review also covers the extensive literature on the application of PLGA in delivering small-molecule drugs, proteins, peptides, antibiotics, and antiviral drugs. Furthermore, we discuss the role of PLGA-based DDSs in the treating various diseases, including cancer, neurological disorders, pain, and inflammation. The incorporation of drugs into PLGA nanoparticles and microspheres has been shown to enhance their therapeutic efficacy, reduce toxicity, and improve patient compliance. Overall, PLGA-based DDSs holds great promise for the advancement of the treatment and management of multiple chronic conditions.

## 1. Introduction

Drug delivery systems (DDSs) are designed to deliver drugs to specific target sites, offering many advantages, including enhanced therapeutic efficacy, improved patient compliance, reduced dose-related toxicity, and support for a broader range of medical treatments [1]. The routes of DDSs vary depending on the specific treatment, whether oral, pulmonary, injectable, or transdermal, and implantation method [2].

Controlled drug delivery (CDD), which can be categorized into passive and active-controlled DDSs, releases drugs at a predetermined rate and for a predetermined duration to maintain therapeutic drug levels in the body while improving efficacy and minimizing side effects [3,4]. Compared to traditional DDSs, CDD systems are particularly beneficial for drugs with narrow therapeutic windows that require frequent monitoring and adjustment or for long-term treatments [5]. Long-acting drug delivery systems (LADDSs) continuously release active pharmaceutical ingredients (APIs) over periods ranging from weeks to years, revolutionizing the management and treatment of multiple chronic conditions, including cancer [6], neurological disorders [7], pain [8], inflammation [9], and other diseases [10]. LADDSs also offer the benefits of reducing the frequency of injections, minimizing administration pain [11], and improving patient comfort [12].

Various materials are used for formulations to achieve long-acting delivery and controlled drug release, such as homopolymers of poly(lactide) or poly(lactic acid) (PLA), polyester copolymers of lactide and glycolide (PLG), and copolymers of lactic acid and glycolic acid (PLGA) [13]. The molecular weight and monomer ratio of these polyester copolymers play a crucial role in determining the duration of sustained release time. PLGA, which is widely used due to its excellent biocompatibility and biodegradability, is commonly utilized in DDSs. PLGA-based DDSs can be tailored to release drugs at controlled rates, protect them from degradation, and enhance stability throughout the product life cycle [14]. PLGA can be synthesized through direct condensation of lactic acid and glycolic acid or by ring-opening polymerization (ROP) of lactide and glycolide cyclic dimers. Initiators like tin (II) bis (2-ethyl hexanoate) (Sn (Oct)_2_), zinc proline, and stannous octoate are used in ROP to obtain PLGA copolymers of the desired weight with hydroxyl and carboxyl end groups [15,16,17]. Meanwhile, PLGA can be modified with functional polymeric blocks such as polyethylene glycol (PEG) [18].

**Figure 1:**
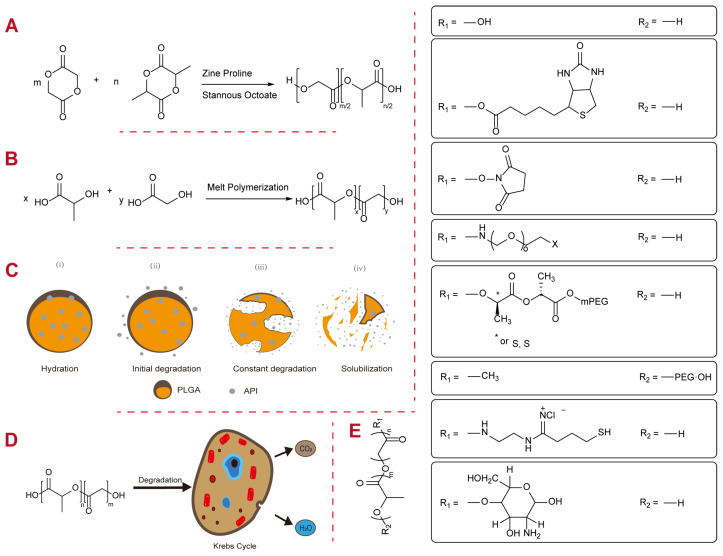
The synthesis and degradation of PLGA. (**A**) The ROP of lactide and glycolide. Source: [17]. (**B**) Direct melt condensation polymerization. Source: [15]. (**C**,**D**) Schematic diagram of PLGA in different degradation stages. Source: [48]. (**E**) The chemical structure of PLGA with different end caps (the asterisk indicates S,S stereoisomer).

In an aqueous environment, PLGA degradation occurs due to multiple factors, including the hydrolysis of ester linkages, decreased molecular weight, and heterogeneous erosion [19]. There are four stages of degradation, namely (i) hydration, (ii) initial degradation, (iii) constant degradation, and (iv) solubilization, as shown in Figure 1. Initially, water penetrates the polymeric matrix, disrupting van der Waals forces and hydrogen bonds, leading to a decrease in the glass transition temperature (Tg). In the initial degradation stage, covalent bonds are cleaved, reducing molecular weights. This is followed by constant degradation, characterized by autocatalysis of the carboxylic end cap and significant cleavage of backbone covalent bonds, leading to mass and integrity loss. Finally, in the solubilization stage, the polymer fragments are cleaved into smaller molecules, allowing the PLGA to dissolve in the aqueous environment [20]. Lactic acid and glycolic acid are considered the products of PLGA biodegradation and are eliminated from the body through the Krebs cycle. Lactic acid is converted into carbon dioxide and pyruvate, which enter the Krebs cycle, while glycolic acid is either excreted in urine or oxidized to glyoxylate, which is also converted into pyruvate and further processed in the Krebs cycle. This results in the production of carbon dioxide and water, which are safely secreted, minimizing systemic toxicity [21,22,23].

Various biodegradable devices and pharmaceutical drug delivery products utilize PLGA, including microparticles (MPs), nanoparticles (NPs), implants, and micelles [1]. MPs, which include spherical microspheres, are larger particles made from various materials and range in size from 1 to 1000 micrometers. They are commonly used for drug delivery, tissue engineering, and other applications [24]. NPs, on the other hand, are small particles in the nanometer range with various shapes, such as rods, cubes, and tubes [25]. Nanofibers, a type of NP, are elongated, thin fibers, while nanospheres are spherical nanoparticles with a high surface area-to-volume ratio, allowing for effective drug loading [26]. Implants are devices inserted into the body and commonly used in orthopedics, cardiology, and other medical fields for sustained drug delivery or structural support [27]. Micelles are self-assembling structures composed of amphiphilic molecules and are commonly used to encapsulate and solubilize hydrophobic drugs within the core and release them in a controlled manner [28].

PLGA serves as both a drug carrier and as the structural framework for drug-loaded materials [29]. In recent decades, the Food and Drug Administration (FDA) has approved many drug delivery products that have become some of the most successful complex drugs on the market. PLGA-based drug carriers have been particularly effective due to their high encapsulation efficiency, superior biocompatibility, stable release performance, and sufficient bioavailability [16]. Additionally, PLGA-based DDSs, particularly those using nano-PLGA, enhance aqueous solubility, bioavailability, and the stability of APIs [30].

Currently, PLGA-based DDSs are being developed to deliver small-molecule drugs, vaccines, proteins, and peptides. Since the introduction of the Lupron Depot^®^ (leuprolide) in 1989, the FDA has approved several PLGA-based drug products, including Risperdal Consta^®^ (risperidone) and the peptide-containing microspheres of Lupron Depot^®^ (leuprolide), as shown in Table 1 [31,32]. Moreover, clinical trials have shown promising results by exploring new indications for existing products or investigating potential new therapeutics. Several studies [33,34] have summarized the various phases of ongoing PLGA-based clinical trials registered at *ClinicalTrails.gov* [35]. For example, a study on naltrexone-loaded microspheres (marketed as Vivitrol^®^) showed approximately 33.70 wt% drug loading and 66.30% of the formulation containing 75:25 PLGA in each vial [36]. In comparison, the commercial Lupron Depot^®^ formulation has a leuprolide acetate loading of 10% and a gelatin loading of 1.7%, with the aliphatic ester end-capped and a 75:25 ratio of PLGA [37].

In recent decades, significant advancements in the continued research and development of PLGA-based formulation technologies have demonstrated innovation and potential to improve therapeutic outcomes and address complex healthcare challenges. This work discusses the latest research on PLGA applications. PLGA nanoparticles can improve drug stability, enhance drug targeting, and provide controlled release profiles for drug delivery applications. Innovative methods for PLGA nanoparticle formulation, such as emulsion solvent evaporation, nanoprecipitation, and electrospraying, are being explored and have shown outstanding outcomes [38,39]. Additionally, stimuli-responsive PLGA formulations that respond to specific triggers, such as changes in pH and temperature, have been investigated to reduce side effects and enhance the therapeutic efficacy of drug targeting [40,41,42,43]. The development of PLGA-based delivery systems for combination therapy, which co-encapsulates multiple drugs or therapeutic agents in a single formulation, is another research focus aimed at improving treatment outcomes by targeting multiple pathways or addressing different aspects of a disease simultaneously [44]. Researchers are also exploring the development of hybrid DDSs that combine PLGA with other polymers or biomaterials, such as PEG and chitosan, to enhance the targeted properties and drug delivery performance of the formulations [45]. These hybrid systems offer synergistic effects for the delivery of drugs to the target sites, such as the brain, improving biocompatibility and tailoring drug release profiles. This approach has the potential to reduce drug resistance and improve treatment outcomes [46,47].

## 2. Physicochemical Properties of PLGA

PLGA, a copolymer of lactic acid and glycolic acid, is synthesized through ring-opening polymerization [17,49] of the corresponding lactic acid dimers and glycolic acid dimers or by direct polycondensation of lactic acid and glycolic acid. Upon degradation, it breaks down into carbon dioxide and water, as illustrated in Figure 1 [19]. These copolymers are available with various key properties, including molecular weight, intrinsic viscosity, monomer ratio (L: G ratio), blockiness, and end caps, all of which influence degradation periods ranging from months to years. The critical quality attributes of drug delivery formulations, such as particle size (or distribution) and porosity, exhibit significant differences that affect both in vitro and in vivo performance [50]. It is well known that PLGA characteristics such as a higher molecular weight [51], a higher monomer ratio [52], lower blockiness [53], and acid end caps [54,55] contribute to lower hydrophilicity and, consequently, slower drug release. For example, according to the labeling information approved by the FDA, Leuprolide acetate for injectable suspension, marketed under the brand name ELIGARD^®^, is formulated using different copolymers with varying end caps and monomer ratios. ELIGARD^®^ 7.5 mg is composed of 7.5 mg of drug loading and 82.5 mg of a 50:50 copolymer containing a carboxyl end cap, whereas ELIGARD^®^ 22.5 mg includes 22.5 mg of drug loading and 158.6 mg of a 75:25 copolymer with hexanediol.

### 2.1. Molecular Weight

PLGA exhibits a range of molecular weights, spanning from under 10,000 to up to 200,000 g/mol [56]. These molecular weights can be determined using intrinsic viscosity measurements or gel permeation chromatography (GPC) with polystyrene external standards (PESs). However, due to differences in polymer–solvent interactions, PLGA’s molecular dimensions differ from those of polystyrene, leading to potential inaccuracies when using polystyrene standards as a reference. In commercial practice, intrinsic viscosity is frequently employed to estimate the molecular weights of PLGAs. However, this method’s accuracy is limited by the complex relationship between PLGA’s molecular dimensions and the monomer ratio. Consequently, estimating the molecular weights of PLGAs with varying L/G ratios based solely on a standard curve can be challenging [57]. For improved accuracy, multi-angle static light scattering (MALS) is utilized to measure the molecular weights of PLGAs. Compared with the molecular weights obtained via MALS, those obtained by the PES method may differ by up to 70% under certain conditions [57,58]. As the degradation rate increases due to hydrolysis, lower-molecular-weight PLGA is less likely to exhibit hydrophobic characteristics [59]. The molecular weight of PLGA plays a crucial role in determining the drug release kinetics, mechanical properties, stability, and drug-loading capacity of PLGA-based DDSs. Low-molecular-weight PLGA polymers typically degrade more rapidly, resulting in accelerated drug release, often characterized by an initial burst release followed by a sustained release over time [60]. Additionally, low-molecular-weight PLGA polymers tend to have lower mechanical strength and stiffness, which can compromise structural integrity and stability, potentially leading to premature drug release or reduced drug efficacy. Furthermore, lower-molecular-weight polymers may offer reduced drug-loading capacity due to limited space for drug encapsulation, affecting overall drug delivery efficiency.

### 2.2. Intrinsic Viscosity

A crucial aspect of polymer molecular weight is the intrinsic viscosity of PLGA, which is directly correlated with particle size and drug release rate. High intrinsic viscosity in PLGA polymers, which is associated with high molecular weight, leads to larger particle sizes and a slow drug release rate [61]. Additionally, increased intrinsic viscosity reduces the tendency of protein molecules to diffuse through the polymer matrix, thereby enhancing the encapsulation efficiency of proteins/peptides [62]. The higher intrinsic viscosity also imparts greater mechanical strength and stiffness, which contribute to the structural integrity of the DDS, resulting in improved stability over time and a longer shelf life of DDSs [63].

### 2.3. Monomer Ratio

**Figure 2:**
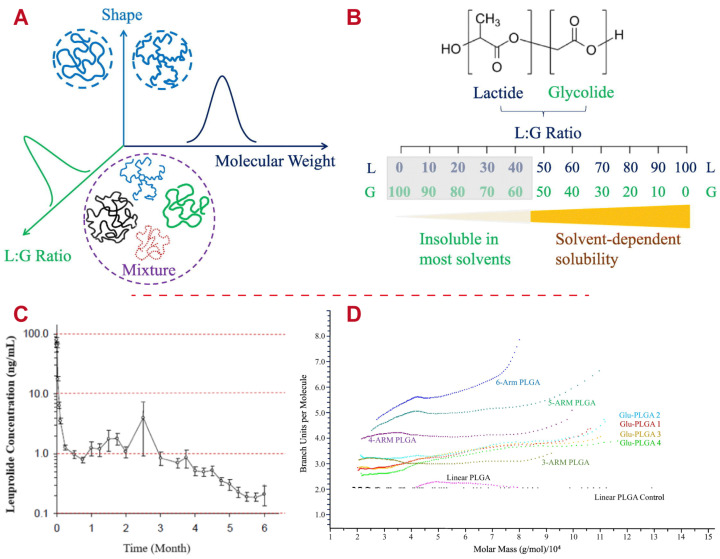
Impact of the PLGA L: G ratio on solubility. PLGAs with varying L: G ratios breaking down in various solvents [57]. (**A**) Characterization of PLGA involves assessing its molecular weight, L: G ratio, and shape, which can be linear or branched. When a formulation contains a mixture of PLGAs with varying properties (as shown in the dotted circle), determining and characterizing each PLGA component becomes difficult. (**B**) PLGAs with varying L:G ratios can dissolve in different solvents, enabling the separation of PLGAs even when their molecular weights are identical. (**C**) The serum leuprolide concentrations in human patients after injection of Eligard, which provides 45 mg of leuprolide for a 6-month effective treatment. (**D**) Assessment of the branch unit numbers in Glu-PLGAs and standard star-PLGAs with 3, 4, 5, and 6 arms. All Glu-PLGAs maintain a 55:45 L: G ratio. Copyright (2019), Elsevier.

The ratio of glycolide and lactide impacts the polymerization and properties of PLGA [64]. For instance, PLGA with a 40:60 ratio indicates that the polymer is composed of 40% lactic acid and 60% glycolic acid. Commonly used PLGA monomer ratios are 50:50 and 75:25 [65]. Due to the higher hydrophilicity of glycolic acid compared to lactic acid, PLGA with a greater glycolic acid content exhibits faster hydrolysis and degradation rates, resulting in a shorter degradation time [66]. As illustrated in Figure 2, degradation decreases with a higher L: G ratio, as the more hydrophobic methyl group leads to gentler absorption and slower water diffusion [57]. The monomer ratio greatly influences the properties of PLGA and is crucial in determining its performance in drug delivery applications. Generally, increasing the glycolic acid content in PLGA results in a more acidic microenvironment and faster degradation due to the higher hydrophilicity of glycolic acid relative to lactic acid. This change accelerates drug release from the delivery system [67,68]. By adjusting the monomer ratio, it is possible to customize the drug release profile to achieve sustained or controlled release of the drug over a desired period. Compared to PLGA with a higher lactic acid content, PLGA with a higher glycolic acid content tends to be more brittle and has lower tensile strength [69]. Therefore, the choice of monomer ratio is critical for applications where mechanical properties are important, such as in implantable devices. Careful selection of the monomer ratio allows researchers to tailor the properties of PLGA-based delivery systems to meet specific therapeutic requirements and improve their efficacy and safety.

### 2.4. Blockiness

The blockiness of PLGA refers to the alternating sequence of lactic acid and glycolic acid units within the polymer chain. This blocky structure imparts unique properties to PLGA, including tunable degradation rates, mechanical strength, and biocompatibility [70].

Research [71,72] indicates that one of the primary mechanisms of drug release from PLGA-based microspheres is hydrolytic degradation in aqueous PLGA media. Studies on microsphere degradation [53] have shown that the molecular weight distribution of PLGA with higher blockiness changes more significantly over time. This is because polymers with higher blockiness exhibit greater cleavage of the G-G linkages in aqueous environments compared to those with lower blockiness. Additionally, the blockiness of PLGA affects the stability of PLGA-based DDSs by influencing resistance to hydrolysis. Block copolymers with well-defined blocks of lactic acid and glycolic acid units generally exhibit greater stability than random copolymers. Due to their heterogeneous distribution of monomer units, random copolymers tend to degrade more quickly, leading to a faster drug release from PLGA-based DDSs [73]. Furthermore, the arrangement of lactic acid and glycolic acid units within the polymer chain affects drug interactions, thereby influencing the drug-loading efficiency and capacity of PLGA-based DDSs [74]. Consequently, blockiness is a crucial factor in designing and optimizing PLGA-based DDSs for specific therapeutic applications.

### 2.5. End Caps

Commercial PLGA typically features two main types of end caps, namely acid end caps and ester end caps, as illustrated in Figure 1. Ester-capped PLGA includes a long alkyl chain, often utilizing decanol or dodecanol as the initiator. In contrast, acid end-capped PLGA is produced by using lactic acid as an initiator and lacks this long alkyl chain [75]. The primary distinction between these two types of end caps is the presence of a long alkyl chain with a terminal methyl in the ester end-caped PLGA. This can be confirmed by detecting a methyl signal at 14 ppm in the ^13^C NMR spectrum for ester end-capped PLGA, while the absence of this peak indicates an acid end cap [75,76].

The choice of end cap influences the characteristics of PLGA microspheres. PLGA with ester end caps generally degrades more slowly compared to those with acid end caps [21]. In addition, there is a positive correlation between the presence of acid end caps and the release rate of hydrophobic drugs [77]. The acid end cap facilitates swelling of the PLGA matrix by initiating hydrolysis due to its water uptake potential. Consequently, among PLGAs with similar molecular weights and L:G ratios, ester end-capped PLGA typically exhibits a delay of four to six weeks in degradation in vivo compared to acid end-capped PLGA [78]. In recent decades, PLGA has been synthesized and modified with various functional groups to create novel end-capped copolymers. These advancements have expanded the applications of PLGA-based DDSs for a variety of purposes [17,18,79,80,81], as depicted in Figure 1.

## 3. Modification of PLGA-Based DDSs

To date, PLGA has been extensively modified to enhance its properties for DDSs. Researchers can choose from a range of modification strategies, depending on the desired outcomes and specific requirements of the delivery system.

Several approaches can be employed to modify PLGA, including surface modification, coating, blending, functionalization, and cross linking. Surface modification can be achieved by conjugating functional groups, such as amino groups or carboxylic acids, to the surface of PLGA nanoparticles. These functional groups enable the attachment of targeting ligands, antibodies, or imaging agents, thereby improving the stability, biocompatibility, or targeting capabilities of DDSs [82]. Coating with other polymers or materials can also modify PLGA surface properties. For instance, coating with PEG can extend the circulation time of PLGA nanoparticles in the body and reduce the immune response. Coating with chitosan or alginate, on the other hand, can enhance the mucoadhesive properties of PLGA, making it suitable for targeted delivery to mucosal surfaces [83]. Blending PLGA with other polymers allows for the creation of hybrid materials with tailored properties. For example, blending PLGA with polyethylene oxide (PEO) can improve the flexibility and mechanical strength of the polymer [84], while blending with PEG can enhance its hydrophilicity and drug release profile [85]. Chemical functionalization of PLGA introduces specific functional groups that can be used for drug conjugation, targeting, or controlled release. For example, functionalizing PLGA with hydrophilic polymers, peptides, or proteins can improve its interaction with biological tissues [86]. Cross linking PLGA nanoparticles can enhance their mechanical properties and stability, which improves drug-loading capacity and enables sustained release profiles. An example is the use of dimethyl dioctadecyl ammonium bromidebe (DDAB) to cross link protein with PLGA, facilitating stable delivery of protein into mitochondria [87].

For example, self-assembly structures of micelles are formed by amphiphilic molecules of dextran with PLGA by covalent bonding. PLGA serves as the hydrophobic copolymer, while dextran forms the hydrophilic shell due to its variable linkages and natural branches. This naturally creates an amphiphilic block copolymer that self-assembles into micelles [88]. Another example involves aspirin-loaded PLGA nanofiber coatings applied to the surface of titanium via electrospinning. These coatings can release aspirin over a period of up to 60 days [89]. Moreover, they promote the proliferation and osteogenic differentiation of bone mesenchymal stem cells while inhibiting M1 polarization and osteoclast differentiation of macrophages in vitro, representing a potential therapy to prevent inflammation and periprosthetic bone loss.

## 4. Load of PLGA-Based DDSs

### 4.1. Small-Molecule Drugs

Risperidone is a small-molecule, atypical antipsychotic agent known for its water-insoluble properties and its use in the treatment of neurological disorders, particularly schizophrenia, where it causes fewer extrapyramidal side effects (EPSs) than other antipsychotics [90]. However, the incidence of EPSs is dose-dependent, making it necessary to administer lower doses over a long-term period for effective management of schizophrenia [91]. To achieve stable and sustained delivery, implantable formulations of risperidone using PLGA have been developed and tested both in vitro and in vivo for pharmacokinetic profiles. These implantable formulations provide stable and long-term delivery of atypical antipsychotics [27]. Research conducted by the team of Dr. Burgess [91] investigated how variations in PLGA characteristics influence the in vitro and in vivo outcomes of risperidone in situ-forming implants (e.g., different molecular weights (Mw), L:G ratios, and end caps). Their findings revealed that even a 5 kDa deviation in molecular Weight (Mw), a 5% shift of the L:G ratio, and differences in the end caps of PLGA altered the release behavior of risperidone in both in vitro and in vivo settings. These results underscore the importance of carefully monitoring and controlling critical quality attributes when developing risperidone in situ-forming implant products. Previous studies [92] successfully established and validated a level-A correlation for risperidone-loaded PLGA microspheres, demonstrating the potential of these formulations in achieving consistent and predictable drug release profiles. For instance, naltrexone, the API in Vivitrol^®^, is used to prepare PLGA microspheres, as it is a water-soluble compound that differs from risperidone. Combining a manufacturing process and dissimilar PLGAs, Vivitrol^®^, has been used to prepare PLGA microspheres due to its water-soluble nature, which contrasts with the water-insolubility of risperidone. The manufacturing process and different PLGA formulations used in creating Vivitrol^®^ result in microspheres that exhibit biphasic release characteristics, showing a high in vitro–in vivo correlation (IVIVC) [93]. Once IVIVC is established, in vitro release methods can serve as surrogate models for bioequivalence studies, potentially reducing the need for extensive animal testing and minimizing the duration and cost of clinical trials [32]. This approach facilitates more efficient drug development processes, particularly for PLGA-based delivery systems designed to release water-soluble APIs with biphasic release profiles.

### 4.2. Natural Product

Paclitaxel (PTX) is a well-known taxane diterpenoid and the best-selling anticancer drug derived from natural sources, marketed under the brand name Taxol. It is widely used in the treatment of breast, lung, and ovarian cancers [94]. However, the clinical efficacy of PTX is limited due to its lipophilic nature, which results in low solubility, non-selective targeting, and rapid clearance from the body, leading to significant side effects [95]. To improve its solubility, formulations of PTX often include solubilized excipients such as Cremophor EL (CrEL) and absolute ethanol. However, these excipients are associated with severe side effects, including allergic reactions, nephrotoxicity, cardiotoxicity, etc. [96]. To address these limitations, paclitaxel-loaded PLGA nanospheres have been developed. These nanospheres utilize biodegradable polymers to provide a slower and sustained release of PTX, significantly enhancing its antitumor effect while reducing side effects compared to traditional formulations [97].

Another natural product, curcumin (CUR), is extracted from the dried rhizome of *Curcuma longa* L. and is widely recognized as a chemosensitizer in cancer therapy [98]. Combining CUR with PTX represents a promising cancer treatment strategy, enabling dosage reduction to minimize side effects while improving therapeutic efficacy. Several clinical studies, including the NCT01490996 project sponsored by the University of Leicester, are investigating the combination of CUR with chemotherapy in patients with inoperable colorectal cancer, with some trials currently in phases I and II [35]. However, both CUR and PTX suffer from poor water solubility and low bioavailability, which can limit their therapeutic effectiveness [99]. To overcome these challenges, nanocarriers are employed to encapsulate and protect CUR and PTX, thereby improving their solubility and chemical stability. This encapsulation strategy enhances the circulation time of these drugs in the body, ultimately improving their therapeutic outcomes [100].

### 4.3. Protein or Peptide

Proteins and peptide drugs (PPDs) are highly specific and potent therapeutic agents in pharmaceutical applications. The controlled delivery and release of these biologics have lots of benefits, including improved efficacy, enhanced accessibility, mitigation of dosing side effects, increased patient convenience, and better adherence to treatment regimens [101]. Various techniques have been developed to prepare nanoparticles for loading with PPDs, such as protein adsorption [102] and coating techniques. These methods open up the possibility of introducing biological ligands into the coatings of peptides and anti-protein microspheres in the future, thereby promoting preferential interaction and uptake into the targeted cells. Microspheres with low-protein-binding surfaces that expose specific biological functions provide a versatile platform for the design of targeted drug delivery systems [103].

For instance, leuprolide acetate PLGA MPs are commonly used in the treatment of various medical conditions, such as prostate cancer, endometriosis, and certain types of infertility. These PLGA MPs are injected at the site of action, where they slowly degrade over time, releasing the drug in a controlled manner. This approach has proven effective in managing symptoms and improving the quality of life of patients with these conditions. In a study by Pro. Burgess [104], after comparison with leuprolide acetate MP formulations with varying physicochemical properties, drug release appeared to be dependent on the molecular weight and the weight distribution of PLGA. Specifically, the initial burst release of the drug increased with a higher proportion of low-molecular-weight PLGA, underscoring the importance of controlling burst release in these formulations.

Furthermore, proteins and peptides are often released from the PLGA matrix following a biphasic release profile characterized by an initial burst phase followed by a gradual drug release phase. This release pattern can be influenced by the interactions between the polymers and the drugs, such as peptides with positive charges loaded into the negatively charged polymers or acylated peptides that from adducts during degradation, which exhibit slow release properties over extended periods [33].

### 4.4. Antibiotics or Antiviral Drugs

**Figure 3:**
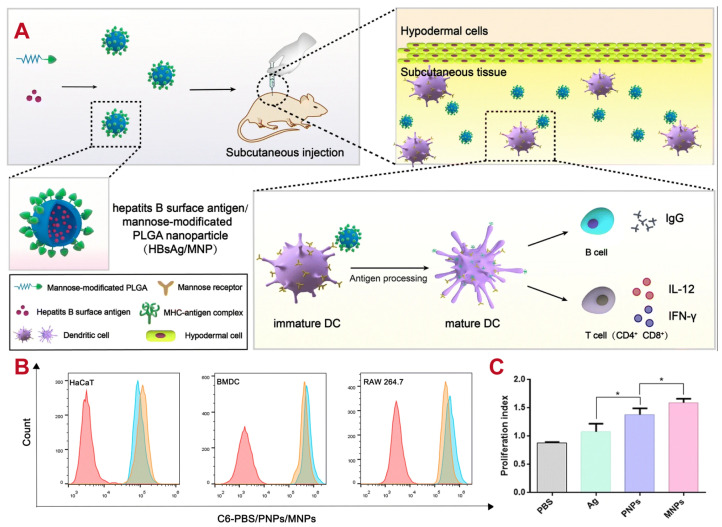
Promising vaccine delivery vehicle: mannose-modified PLGA nanoparticle (MNP)-loaded hepatitis B surface antigen (HBsAg) protein highly presents to lymphocytes, which target and induce long-lasting immunity. (**A**) Schematic representation of the in vivo fate of nanoparticles. (**B**) Flow cytometry histogram of in vitro cellular uptake in HaCaT, BMDCs, and RAW 264.7 cells. Data are presented as mean ± SD (n = 3). * *p* < 0.05. (**C**) Splenocyte proliferation after in vitro co-incubation with Ag [105]. Copyright (2019), Springer Nature.

Antigen-presenting cells beneath the epidermis can be effectively targeted using mannose-modified PLGA nanoparticles (MNPs) loaded with the hepatitis B surface antigen (HBsAg) protein. This approach has been shown to induce potent adaptive immunity responses by enhancing antigen uptake by immune cells [105]. Due to the properties of PLGA, long-lasting immunity can be established through the slow release of HBsAg, which allows for sustained presentation to lymphocytes. This strategy holds significant potential for future vaccine development, as illustrated in Figure 3. Furthermore, the van der Waals forces conferred by mannose modification increase the affinity for proteins, while glycosylated PLGA can improve antigen stability upon release. Overall, mannosylated PLGA NPs represent a promising research direction in terms of increasing delivery efficiency and specificity to targeted cells by inducing the receptor-mediated endocytosis of NPs.

Prodigiosin (PG) is a bioactive red pigment produced through microbial fermentation of Serratia marcescens strains, as well as other microorganisms. Being an effective proapoptotic agent against various cancer cells, including those with multidrug resistance cells, PG demonstrates its superiority with minimal toxicity on normal cells [106]. The delivery of PG to breast tumors using PLGA-based microparticles was studied with paclitaxel serving as a control [107]. The research demonstrated that PG-loaded PLGA microparticles, which release PG at controlled rates, significantly reduce the viability of MDA-MB-231 breast cancer cells by halting mitosis and inducing apoptosis. Additionally, a clinical study was initiated in 2021 (NCT04735601), investigating Ahmed valve implantation with mitomycin C-coated PLGA for the treatment of Sturge Weber syndrome glaucoma in adults. This study is in Phase III, although results have not yet been posted [35].

## 5. Applications of PLGA-Based DDSs

### 5.1. Pain

Severe side effects may result from repeated use of systemic administration of analgesic drugs. In contrast, persistent pain can be treated with or without reduced side effects using local anesthetics (LA; e.g., ropivacaine [RVC]). However, the effect of local anesthetics after a single injection lasts only several hours. Postoperative pain may last for a few days, several months, or even more than 1 year. Therefore, producing a prolonged analgesic effect of new LA formulations is a compelling need. Sustained-release RVC (SRR) and its effect on postsurgical and neuropathic pain can be observed using RVC-loaded PLGA particles. The administration of local SRR [108] has been shown to have a long-lasting antinociceptive effect on neuropathic pain with biosecurity and tolerance by the body.

**Figure 4:**
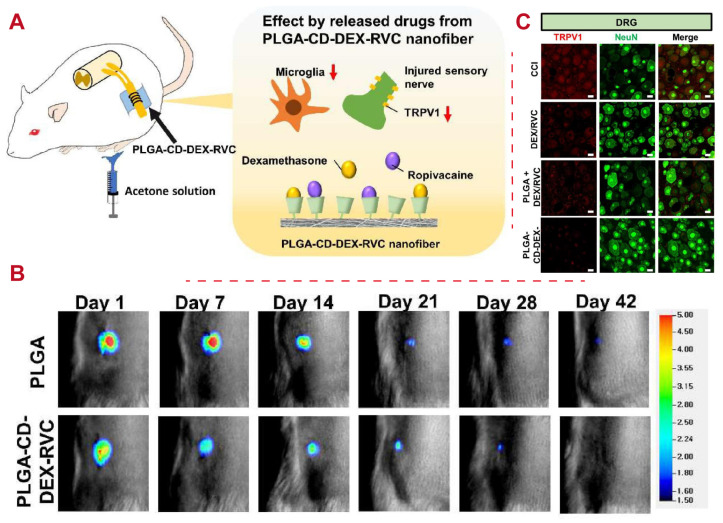
An example of PLGA application for pain treatment. (**A**) Schematic drawing of in vivo experiments on Sprague–Dawley (SD) rats with chronic constriction injury (CCI). PLGA-CD-DEX-RVC nanofibers were implanted into the sciatic nerve and measured by an acetone test to confirm the cold allodynia response. The mitigating mechanism of neuropathic pain is blocking of the transient receptor potential vanilloid 1 (TRPV1) channel and inhibition of microglia. (**B**) Luminescence imaging after transplantation of the PLGA and PLGA-CD-DEX-RVC nanofibers. (**C**) Immunofluorescence staining of TRPV1 and NeuN in DRG. Scale bar: 20 μm [8]. Copyright (2022), Elsevier.

The epidural space of the spine can be injected with an anti-inflammatory agent dexamethasone (DEX) and local anesthetic ropivacaine (RVC) in clinical settings to treat neuropathic pain in patients. However, uncontrolled flow of short-acting drugs to the motor nerve is observed in association with such drug treatment [8]. Therefore, PLGA nanofibers containing DEX and RVC (PGLA-CD-DEX-RVC nanofibers) have been developed to overcome these limitations. The nanofiber scaffold enables many drugs to onboard the surface. Furthermore, synthesized PLGA-CD-DEX-RVC nanofibers release both drugs sustainably for over 48 h and relieve allodynia cold sensitivity consistently for 14 days. Moreover, these nanofibers can prevents drugs from entering motor nerves, as shown in Figure 4. PLGA-CD-DEX-RVC nanofibers are a good longer-term treatment option for neuropathic pain in clinical settings.

### 5.2. Cancers

**Figure 5:**
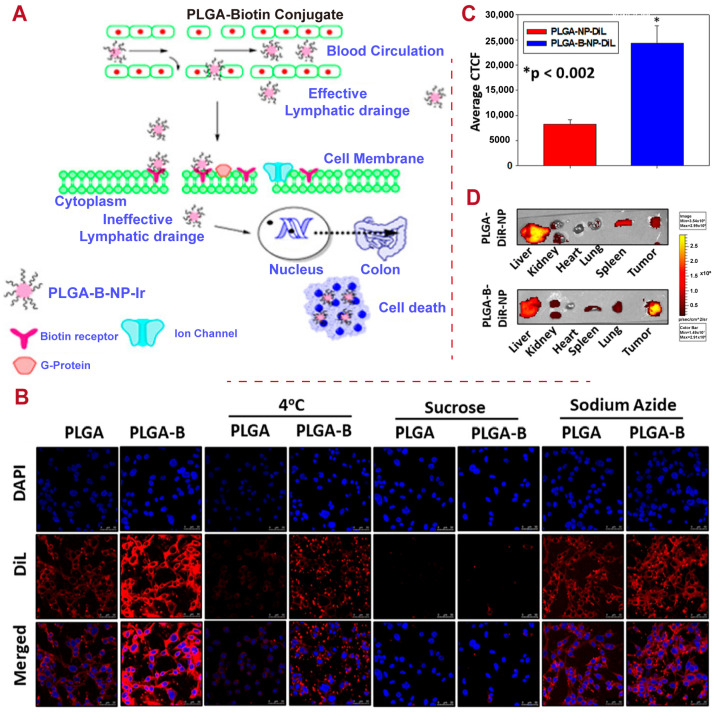
An example of PLGA application for cancer treatment. (**A**) Schematic illustration of the mechanism whereby PLGA–biotin conjugate irinotecan nanoparticles target colon cancer. (**B**) In vitro cellular uptake study followed by confocal analysis. (**C**) Corrected total cell fluorescence of Cy5.5 quantified from images using NIH ImageJ software (ImageJ Freeware; https://imagej.net/ij/, 8 October 2023) and represented in a bar graph (mean ± SEM). (**D**) Near-infrared fluorescence (NIRF) images of the tumors and major organs dissected from PLGA-NP-DiR and PLGA-B-NP-DiR-treated mice. Source: [17].

Photothermal therapy (PTT) is a treatment method that uses light-absorbing agents to produce heat upon exposure to laser light. Targeted heating can effectively pinpoint and eliminate cancer cells or other unhealthy tissues while preserving the integrity of surrounding healthy tissue. Utilizing PLGA as a carrier for light-absorbing agents (e.g., gold nanoparticles or organic dyes) allows for controlled release at the designated target site upon laser light activation and reduces systemic toxicity, making it a promising approach for the safe and effective long-term treatment of various diseases [109]. PLGA loaded with black phosphorus quantum dots (BPQDs) can produce biodegradable BPQD/PLGA nanospheres with oil-in-water emulsion solvent evaporation. The hydrophobic PLGA prevents their rapid degradation by isolating the interior BPQDs. After intravenous injection, the NSs boast long blood circulation in the body through the EPR effect, which enhances photothermal stability and efficient tumor aggregation for efficient photothermal cancer treatment. BPQDs/PLGA NSs can be degraded to nontoxic, biocompatible products and cleared in a certain period after completing the therapeutic functions [110]. A biotin-conjugated PLGA polymer for irinotecan nanoparticles (PLGA-B-NP-Ir), as shown in Figure 5, can improve the solubility of the drug after biotin conjugation and advance active targeting, as well as the EPR effect, leading to significantly delayed tumor growth after multiple systemic injections in a mouse model [17]. This suggests the potential for active targeted drug delivery applications for cancer treatment.

Autophagy inducers improve tumor-cell sensitivity to chemotherapeutic drugs and boost antitumor efficacy [111]. Thus, the use of a combination of autophagy inducers and anticancer drugs is a potential strategy for cancer therapy. According to the results of Zhang’s study, autophagosomes can be degraded into lysosomes by PLGA-based NPs, inducing autophagy; therefore, autophagy inhibitors were combined with drug-loaded NPs. The NPs from endosomes and lysosomes causing NPs can be degraded by autophagy inhibitors to sustain existing drug release in the cytoplasm. In addition, autophagy inhibitors can block the autophagy from cancer cells to resist chemotherapeutic drugs [112]. Docetaxel with PLGA NPs, together with autophagy inhibitors (e.g., 3-MA and CQ), can greatly boost the both in vitro and in vivo therapeutic outcomes [113].

Surface modification with PEG can shift PLGA-based NPs with negative charges to neutral or positive values [114]. PLGA NPs endowed with the ability to escape from the endo-lysosome have many potential applications because lysosomes are the drug target, although they should be avoided for the degradation of PLGA-based NPs and more efficient drug delivery [115]. Furthermore, surface modification can enhance the biocompatibility of hydrophobic PLGA NPs. Such a modification can be performed by hydrophilic molecules, such as PEG and d-a-tocopheryl polyethylene glycol 1000 succinate (a water-soluble derivative of natural vitamin E and TPGS). This may be because PEG and TPGS-modified NPs are more easily recognized by and induce autophagosomes [113]. Additionally, the pre-existence of cancer-specific cytotoxic T lymphocytes (CTL) at the tumor site can be achieved by effectively treating immune checkpoint blockade. The authors of [116] studied PLGA particle-elicited CTL responses to multiple human tumor peptide antigens of various cancers.

### 5.3. Neurological Disorders

Pharmacotherapy for central nervous system disorders is complicated, as the BBB and extensive first-pass metabolism impede most chemical and biological drugs. Thus, it leads to low therapeutic efficacy and aggravated side effects caused by dose increase and drug accumulation [117]. Adsorptive-mediated transcytosis (AMT) provides a pathway for drug transport to the brain targeted across the BBB [118]. A mannose coating over PLGA NPs for delivery of Donepezil (DPZ) and Memantine (MEM) holds excellent prospects for the treatment of Alzheimer’s disease. Mannose-coated PLGA NPs are targeted for effective brain delivery [105], as ligand coating provides improved targeting with AMT [119], as applied in copolamine-induced a murine model via the intranasal (IN) route [120]. This could ferry biologic drugs effectively to the brain by overcoming the drawbacks of oral and intravenous routes [121]. Mannose-coated PLGA NPs not only provide excellent targeting and improved brain delivery but also offer maximum bioavailability of both drugs in the brain and minimize exposure to peripheral organs [120].

Curcumin, a hydrophobic polyphenol medication known for its remarkable pharmacological effects on the nervous system, potentially alleviates neurological disorders [122]. However, the clinical application of curcumin is hampered by various factors, such as poor solubility, low permeability, low stability in the body fluids under low aqueous solubility, limited bioavailability, rapid clearance, and reduced absorption in the gastrointestinal (GI) tract [123,124]. PLGA NP-based delivery systems enhance the aqueous solubility and bioavailability of hydrophobic curcumin, enabling it to penetrate the BBB and improving its distribution throughout the body. Furthermore, the encapsulation of curcumin in NPs is believed to enhance its stability and protect it from enzymatic and pH degradation [98,122,125].

Consequently, PLGA-based DDSs provide a consistent and convenient medication regimen to help improve neurological patient compliance, leading to better treatment outcomes and improved overall quality of life.

**Figure 6:**
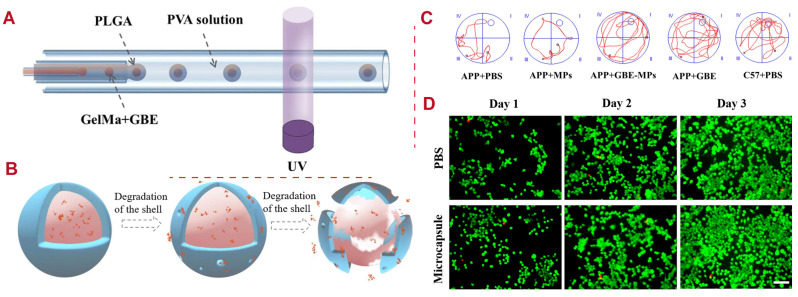
Examples of PLGA applications for neurological disorders. (**A**) Schematic capillary microfluidic chip for preparation of GBE-GelMa-PLGA MPs in double-emulsion droplets. (**B**) Illustration of MP degradation and drug release. (**C**) Search paths of mice in different groups of the Morris water maze (MWM) test. (**D**) Fluorescence images of N2a cells in the control group (blank well) and the experimental group (GelMa-PLGA MPs) taken using a fluorescence microscope. Source: [126].

Microcapsules with gelatin methacrylate cores and PLGA shells (GelMa-PLGA MPs) are designed using a capillary microfluidic technique for encapsulated ginkgo biloba extract (GBE) delivery, as shown in Figure 6, as the low bioavailability and short half-life of GBE in vivo limit their neurological application. GBE is slowly released from solidified microcapsules during PLGA degradation and shows good biocompatibility in cell experiments. When GBE-containing GelMa-PLGA MPs are applied to APP/PS1 mice, amyloid deposition is reduced in the mouse brain, and cognitive impairment is improved. These results indicate that GBE-encapsulated MPs have potential clinical value in treating AD and other neurodegenerative diseases [126].

### 5.4. Inflammation

Osteoarthritis (OA) is the most common chronic joint disorder caused by degeneration or loss of articular cartilage. However, articular cartilage damage that occurs during OA progression cannot be treated. Tetramethylpyrazine (TMP) has strong anti-inflammatory and chondroprotective activities but is rapidly cleared from the joint cavity after joint injection. Thus, multiple injections are required to maintain the efficacy of treatment. Encapsulation of TMP in PLGA microspheres enhanced TMP retention within the joint for 30 days, reduced the number of injections, and decreased dosage from 12.6 mg to 4.6 mg compared with a TMP solution. Joint injection of TMP microspheres efficiently relieves inflammatory symptoms, improves joint lesions, and decreases chondrocyte depletion [127].

**Figure 7:**
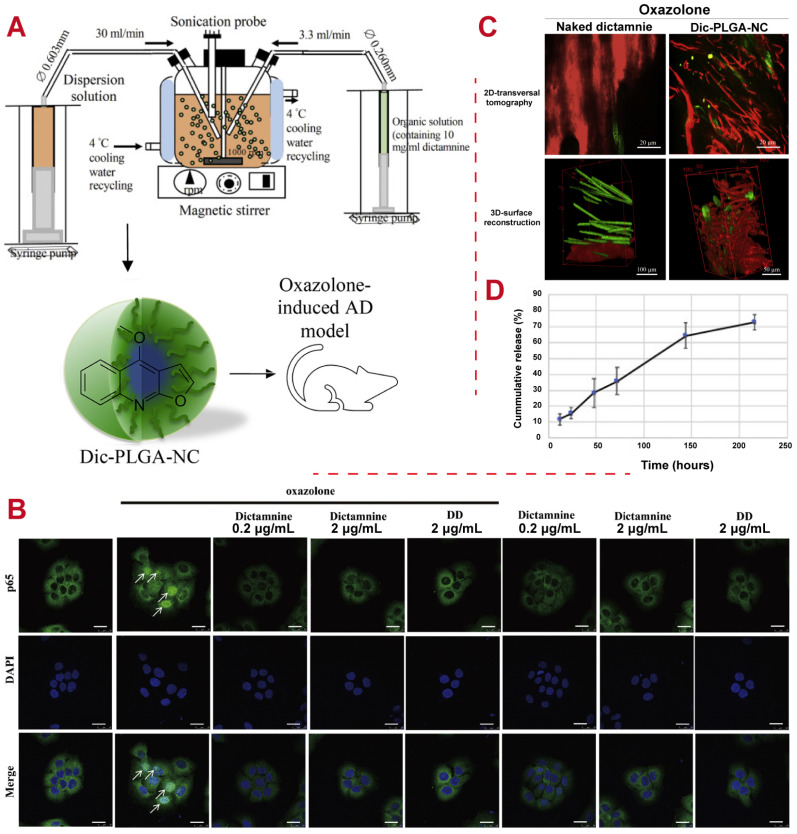
Examples of PLGA applications for the treatment of inflammation. (**A**) Schematic representation of the manufacturing process and animal model of Dic-PLGA-NC. (**B**) Representative confocal microscopic images showing cellular localization of endogenous p65 in oxazolone-stimulated HaCaT cells (arrows) with dicatmnine treatment. DD, dictamnus dasycarpus. Scale bar: 25 μm. (**C**) Representative images showing two-photon microscopic evaluation of mouse skin; this demonstrates that the nanoformulated dictamnine reached the dermal tissue layer and possessed superior skin penetration capability for AD therapy relative to naked dictamnine. (**D**) Dic-PLGA-NC cumulatively released over 216 h in PBS [128]. Copyright (2021), Elsevier.

Dictamnine (i.e., dictamnus dasycarpus) is an active pharmaceutical ingredient in Chinese herbal medicine that is commonly used to treat skin inflammation like atopic dermatitis. PLGA has been used as encapsulated dictamnine drug nanocarrier (Dic-PLGA-NC) to study anti-inflammatory mechanisms. Compared to naked dictamnine, Dic-PLGA-NC was found to improve the drug release rate and penetrate more efficiently into epidermal structures of a dermatitis mouse model induced by oxazolone, as shown in Figure 7, which may be attributed to the controlled, sustained release from PLGA carriers [128].

A monocyte membrane-coated, PLGA nanoparticle-based, gliclazide-loaded nanoghost was developed to control inflammation and atherosclerosis [129]. A nanoghost (NG) is a novel bioinspired, nanoscale vehicle with an increased half-life that escapes the immune response through cell membrane-coated surface functionalization.

### 5.5. Vaccines

In recent years, drug delivery technologies have been developed to improve human health in multiple ways, including by enhancing therapeutic delivery to target sites, minimizing off-target accumulation, and improving patient compliance [130]. Compared to traditional prime-boost regimes, single-dosing vaccines offer significant benefits with increasing vaccine population coverage, reducing costs and saving time, as patients only require one health check [131]. Furthermore, a single-dosing vaccine can rapidly induce a protective immune response, which is preferred under pandemic conditions. Still, many vaccines require more than one dose to fully protect the host at present [132].

A single-dose influenza vaccine derived from recombinant outer membrane vesicles (rOMVs) was designed as PLGA microparticles displaying an antigen-mapped heterospecies tandem sequence of influenza virus M2 protein for release over a month. The protective immune response is long-standing and elicited sustained antibody titers for six months after initial vaccination and 100% survival of mice severely infected with influenza [132]. Antigens and adjuvants encapsulated in PLGA lead to increased cellular response, partly because of enhanced antigen presentation by macrophages and dendritic cells (DCs) [133]. As the ability of dendritic cells to engulf depends on the particle size [134], determination of the ideal size is necessary in future works involving rOMVs within PLGA MPs in order to achieve a long-lasting, optimized, and potent cellular response. A multi-compound particulate vaccine targeting DCs was formulated as PLGA-based NPs to transport Ag protein and adjuvants via type I transmembrane protein CD40, intending to stimulate efficient cytotoxic CD8+ T-cell responses [135].

Restricted by it limited bioavailability and poor immunogenicity, subunit antigen vaccination is unstably successful, and soluble antigens are often unsuccessful when cross-presented. DDSs formulated with PLGA can overcome these problems by protecting antigens from degradation and increasing biodistribution to improve the ability to be uptaken by antigen-presenting cells (APCs) [136].

**Figure 8:**
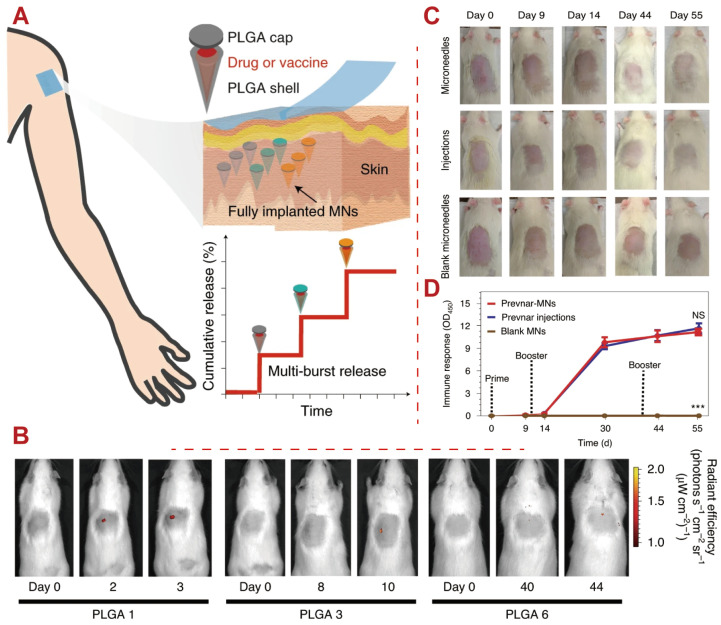
Examples of PLGA applications for vaccine development. (**A**) Schematic illustrations of fully implanted core-shell microneedles (MNs) that deliver their payloads in pulses to mimic drug release from multiple bolus injections over a long period. (**B**) Optical IVIS analysis images demonstrating the pulsatile release of the core-shell microneedles embedded inside the skin of rats over a long period. (**C**) Optical images of the skin of rats from groups that received Prevnar-13 core-shell MNs, s.c. injections, and blank MNs. (**D**) The immune response of rats that received the Prevnar-13-loaded core-shell MNs, multiple bolus s.c. injections, and blank MNs. Data are presented as mean ± SEM; n = 5 rats per group; independent experiments. Statistical analysis was performed using a two-way ANOVA with repeated measures; *** *p* < 0.001; NS, adjusted *p* = 0.7973 [137]. Copyright (2020), Springer Nature.

Using a 3D manufacturing process, a transdermal PLGA core-shell microneedle patch was developed by tailoring PLGA degradation to precisely control drug release. The PLGA cores and shells enable the release of the vaccine payload in pre-programmed bursts over several days to over a month in a single administration. In the clinical use setting, multiple PLGA core shells with varying degradability kinetics settings in microneedles use skin a single injection for several burst releases across multiple periods. In rats, the use of Prevnar-13 in loaded microneedles for the bacterium *Streptococcus pneumoniae* caused similar immune responses as those to the bacterium *Streptococcus pneumoniae* with multiple bolus injections, as shown in Figure 8. Thus, pre-programmed microneedle patches offer alternative strategies for prevention and treatment regimens requiring multiple injections [137]. Such strategies may overcome the scalability and cost-effectiveness issues associated with producing large quantities of PLGA-based vaccines at a reasonable cost.

### 5.6. Tissue Regeneration

In regenerative medicine, PLGA has received recognition as a biological substitute, since the FDA approved its clinical use for restoring, maintaining, or improving damaged tissues [22]. PLGAs have been developed as regenerative medicine delivery carriers in some tissue regeneration and remodeling fields, such as skin, including wound healing, articular cartilage, bone regeneration, and muscle and neural remodeling [138,139,140,141,142]. However, long-term safety, encapsulation efficacy, inflammation, and immune reactions should be considered during regeneration treatment. Furthermore, 3D printing technology has been increasingly used to fabricate complex PLGA scaffolds that can mimic the structure and properties of native tissues, providing a platform for cell growth and tissue regeneration.

**Figure 9:**
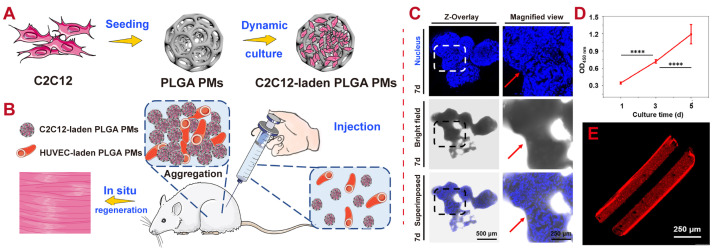
Examples of PLGA applications for tissue regeneration. (**A**) Schematic illustration of the construction of C2C12-laden PLGA PMs by dynamic culture method. (**B**) Evaluation of the in vivo myogenic performance of minimally invasively injected composite microtissues in severe combined immunodeficiency (SCID) mice. (**C**) CLSM images showing the formation of C2C12 aggregation with prolonged culture time (7 d). (**D**) CCK-8 assay assessing the numbers of HUVECs encapsulated in the vessel-like PEG HMs. **** *p* < 0.0001. (**E**) CLSM images showing the longitudinal cross-section of PEG HMs without cells [141]. Copyright (2021), Elsevier.

A unique combination of two minimally invasive therapy options involves the injection of modular microtissues. These microtissues consist of mouse myoblast (C2C12)-laden PLGA porous microspheres (PLGA PMs) and human umbilical vein endothelial cell (HUVEC)-laden PEG hollow microrods (PEG HMs), along with an established extracellular matrix (ECM) to facilitate the development of skeletal muscles in their natural locations. This highly porous structure of PLGA PMs can be applied to C2C12 cells to obtain an effective culture. Discrete C2C12-laden PLGA PMs can be constructed in 7 days of incubation, with strong cell–cell co-relation among C2C12 cells, resulting in the connection of the discrete PLGA PMs (denoted with red arrow in the CLSM image in Figure 9). Moreover, vascular analog-laden PEG HMs in muscle-like constructs have shown promising results in stimulating the enhancement of blood vessels after injection and improving newborn vessels [141].

A synthesized glycine–histidine–lysine peptide (GHK) conjugate with L-carnitine was loaded into PLGA NPs to improve wound healing and exert anti-inflammation effects. Even though GHK and L-carnitine play a crucial role in wound tissue regeneration, upon PLGA degradation, a lactic acid pool could accelerate wound closure in synergetic relation [143,144].

## 6. Limitations and Challenges of Using PLGA-Based DDSs

Even though PLGA is generally considered biocompatible, some studies suggest that PLGA may be toxic both in vitro and in vivo because of the presence of residual stabilizing molecules, inconsistent preparations, and batch-to-batch variations, potentially causing adverse effects on tissue health and environmental impacts [145]. Compared to simpler DDSs, PLGA formulations are more complex, requiring additional manufacturing steps and quality control measures, which result in higher manufacturing material costs. On the other hand, long-term PLGA-based DDSs can contribute to their cost savings by reducing the need for device replacement and leading to prolonged therapeutic effects, potentially lowering overall treatment costs. Considering the complexity and cost of production of PLGA-based DDSs on a large scale, ensuring consistent quality and reproducibility across batches is essential for clinical use. PLGA not only exhibits burst release with inconsistent drug release profiles, which may result in an initial overdose followed by a rapid decrease in drug concentration [146], but also a lower drug-loading capacity and stability, which restrict the drugs that can be delivered [147]. It is necessary to ensure the safety of PLGA and its effective drug delivery, including by enhancing the drug-loading capacity and the effect of encapsulation, which is one of the big challenges in developing PLGA-based DDSs. Acidic byproducts degraded from PLGA may also cause tissue irritation and accelerate drug release kinetics, making it difficult to achieve consistent and predictable drug release profiles in clinical settings. Despite extensive preclinical studies, there is still a limited understanding of the in vivo behavior of PLGA-based DDSs, including their pharmacokinetics and biodistribution. Moreover, the lack of standardized methods for evaluating the performance of PLGA-based DDSs is another main challenge, as PLGA is sensitive and can be influenced by environmental factors and its physicochemical properties. It is difficult but important to establish a stable IVIVC drug delivery system for consistent API performance and the development of product formulations. Optimizing novel polymer blends, developing smart stimuli-responsive systems, and investigating advanced drug delivery strategies (e.g., nanotechnology and 3D printing) can potentially further improve PLGA-based DDSs by enhancing drug-loading capacity and control drug release kinetics.

## 7. Conclusions and Future Perspectives

In conclusion, PLGA has emerged as a versatile and promising material for DDSs due to its biodegradability, biocompatibility, and tunable physicochemical properties. Using PLGA in the formulation of drug delivery pharmaceutical products, such as in situ-forming systems, nanoparticles, microspheres, and micelles, has shown significant potential in enhancing the efficacy of treatments and reducing the toxicity of various drugs. This review highlights recent research applications of PLGA to deliver small-molecule drugs, proteins, peptides, and antibiotics or antiviral agents. The applications of PLGA-based DDSs extend across a broad spectrum of diseases, including cancer, neurological disorders, pain, inflammation, vaccines, and tissue regeneration.

Future research should focus on optimizing the physicochemical properties of PLGA and exploring innovative strategies for targeted and controlled drug release to expand the clinical potential of PLGA-based DDSs further. Firstly, the development of PLGA in sustained-release systems for pain relief medications is both necessary and urgent, particularly in the context of rising concerns over drug abuse [148]. To achieve successful LADDS products, it is crucial to address challenges such as the initial burst release and subsequent incomplete release. Secondly, while novel PLGA NP-based vaccine technologies have become a research trend, they require further refinement, particularly in increasing drug load concentrations for human participants. This could lead to the development of long-acting vaccine delivery systems that provide targeted long-term immunotherapy for cancer treatment [149]. However, there still are various challenges that need to be solved, including the need for real-time monitoring of the in vivo fate to ensure the safety and ability of NPs to carry smaller genes for targeted delivery. Thirdly, surface-modified PLGA scaffolds hold promise for the loading of stem cells and immune cells for applications in tissue generation [150], inflammation, and neurological treatments [151]. Given the complexity of formulation development, it is necessary to establish effective IVIVC models to minimize time and costs and reduce reliance on animal and human studies. This involves the detailed exploration of the physicochemical properties that influence PLGA degradation rates, the development of predictive in vitro release tests, and reliable in vivo pharmacokinetic studies.

## Figures and Tables

**Table 1:** List of commercial PLGA drug delivery products approved by the FDA.

Product name, Manufacturer	API	Type	Approbsl Date(s), Indication(s)	Dose
Vivitrol^®^ Alkermes, Inc., Dublin, Ireland	Naltrexone	Microparticle	Alcohol dependence, relapse in opioid dependence	380 mg per month
Zoladex^®^ Depot AstraZeneca, Cambridge, UK	Goserelin acetate	Implant	Breast cancer, prostate cancer, endometriosis	3.6 mg/10.8 per month
Lupron Depot^®^ AbbVie Inc., North Chicago, IL, USA	Leuprolide acetate	Microsphere	Endometriosis, advanced prostate cancer	7.5 mg per month
Lupron^®^ AbbVie Inc., North Chicago, IL, USA	Leuprolide acetate	Microsphere	Endometriosis	3.75 mg per month
Eligard^®^ Tolmar, Inc., Fort Collins, CO, USA	Leuprolide acetate	In situ gel	Prostate cancer symptoms	7.5 mg a month 22.5 mg per 3 months 30 mg for 4 months 45 mg per 6 months
Sandostatin^®^ LAR Novartis, Basel, Switzerland	Octreotide acetate	Microsphere	Acromegaly, flushing episodes and watery diarrhea (caused by vasoactive intestinal peptide tumors)	10/20/30 mg per month
Atridox^®^ Atrix, Inc., Fort Collins, CO, USA	Doxycycline hyclate	In situ gel	Chronic periodontitis.	42.5 mg per week
Nutropin Depot^®^ Genentech, Inc., South San Francisco, CA, USA	Somatotropin	Microparticle	Growth failure, growth hormone deficiency	13.5/18/22.5 mg per month
Trelstar^®^ Tolmar, Inc., Fort Collins, CO, USA	Triptorelin pamoate	Microparticle	Advanced prostate cancer relief treatment	3.75 mg a month 11.25 mg per 3 months 22.5 mg for 6 months
Somatuline^®^ Depot Ipsen, Inc., Basking Ridge, NJ, USA	Lanreotide	Microparticle	Acromegaly, symptoms caused by neuroendocrine tumors	60 mg per month
Arestin^®^ OraPharma, Inc., Warminster, PA, USA	Minocycline HCl	Microparticle	Periodontitis	1 mg per 2 weeks
Risperidal^®^ Consta Janssen, Inc., Titusville, NJ, USA	Risperidone	Microparticle	Schizophrenia	12.5/25/37.5/50 mg per 2 weeks
Perseris™ Indivior Inc., Richmond, VA, USA	Risperidone	In situ gel	Schizophrenia	90/120 mg per month
Ozurdex^®^ Allergan, Dublin, Ireland	Dexamethasone	Implant	Macular edema, diabetic macular edema, non-infectious uveitis	0.7 mg variable dosing frequency
Propel^®^ Intersect ENT, Inc., Menlo Park, CA, USA	Mometasone furoate	Implant	Chronic sinusitis	0.37 mg per month
Bydureon^®^ AstraZeneca, Cambridge, UK	Exenatide	Microparticle	Type 2 diabetes mellitus	2.0 mg per week
Signifor^®^ LAR Novartis, Basel, Switzerland	Pasireotide	Microparticle	Alcohol dependence, relapse in opioid dependence	380 mg per month
Zilretta^®^ Flexion, Inc., Burlington, MA, USA	Triamcinolone acetoamide	Microparticle	Osteoarthritis	32 mg per 3 months
Sublocade™ Indivior Inc., Berkshire, UK	Buprenorphine	in situ gel	Moderate-to-severe opioid use disorder	100/300 mg per month

## Data Availability

Not applicable.
